# Trophic Change and Community Decline in Acrobat Ants After Rainforest Conversion to Cash Crops

**DOI:** 10.1002/ece3.70694

**Published:** 2024-12-23

**Authors:** Jessica Ehlers, Tamara R. Hartke, Noah Janotta, Amanda Mawan, Rizky Nazarreta, Rizky Desriana, Purnama Hidayat, Damayanti Buchori, Stefan Scheu, Melanie M. Pollierer, Jochen Drescher

**Affiliations:** ^1^ Animal Ecology, J.‐F. Blumenbach Institute of Zoology and Anthropology University of Göttingen Göttingen Germany; ^2^ Centre for Biodiversity Monitoring, Leibniz Institute for the Analysis of Biodiversity Change Bonn Germany; ^3^ Department of Plant Protection Faculty of Agriculture, IPB University Bogor Indonesia; ^4^ Centre for Transdisciplinary and Sustainability Sciences IPB University Bogor Indonesia; ^5^ Centre of Biodiversity and Sustainable Land Use Göttingen Germany

**Keywords:** *Crematogaster*, oil palm, rubber, Southeast Asia, stable isotope analysis, tropical land‐use change

## Abstract

The conversion of tropical rainforests to agriculture causes population declines and biodiversity loss across taxa. This impacts ants (Formicidae), a crucial tropical group for ecosystem functioning. While biodiversity loss among ants is well documented, the responses of individual ant taxa and their adjustments in trophic strategies to land‐use change are little studied. Here, we investigated a collection of > 12,000 acrobat ants (*Crematogaster*) from 14 species collected by canopy fogging in four land‐use systems in Jambi Province, Sumatra, including (1) lowland rainforest, (2) jungle rubber (low impact rubber agroforestry), and monocultures of (3) rubber and (4) oil palm. Abundance‐weighted trophic positions as indicated by stable isotopes of carbon and nitrogen were significantly different between land uses: Lower average, maximum, minimum and range of δ^13^C values in monocultures than in rainforest and jungle rubber indicate a shift of *Crematogaster* towards more plant‐based resources, but may be driven substantially by the “canopy effect” of differential carbon assimilation of leaves between lower and higher canopy. Similar Δ^15^N average, maximum and minimum among the land uses, but a significantly reduced range of Δ^15^N in monocultures, suggest lowered trophic diversity and increased stochasticity of trophic niches in monocultures. In contrast, community decline in *Crematogaster* was very pronounced, with density, richness and rarity in monocultures of rubber and oil palm at a fraction of that in rainforest and jungle rubber. *Crematogaster* communities in oil palm formed a subset of those in rubber, which were a subset of those in jungle rubber, which were a subset of those in rainforest. A notable exception was *Crematogaster ferarii*, which was exclusively found in oil palm. In conclusion, tropical land‐use change induces slight shifts in the trophic structure of *Crematogaster* communities, but massive declines in its density and diversity. This likely affects *Crematogaster‐*driven ecosystem functions in agricultural systems in Southeast Asia.

## Graphical abstract

Acrobat Ants (*Crematogaster* spp.) are numerically and ecologically dominant ants in tropical canopies. Rainforest transformation to rubber and monoculture plantations affects their trophic ecology, which coincides with reduced abundance and richness, and altered community composition.

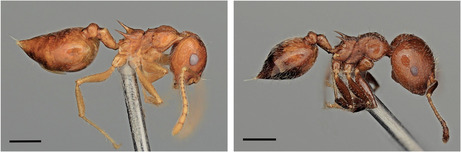

## 1 Introduction

The conversion of natural ecosystems to agriculture is a significant driver of the ongoing biodiversity crisis. This is especially dramatic in species rich tropical ecosystems, where the conversion of lowland rainforest to cash crops may result in species losses exceeding 50% (Drescher et al. 2016; Grass et al. 2020). Ants are among the most abundant arthropods in tropical rainforests (Fayle and Klimes 2022; Schultheiss et al. 2022), often contributing more than half to the specimens in canopy fogging samples (Agosti et al. 2000; Davidson 1997; Pollierer et al. 2023). Due to their abundance and taxonomical and functional richness, ants play important ecological roles in tropical rainforests (Andersen 2000; Davidson et al. 2003; Kaspari 2000), often being referred to as “ecosystem engineers” (Sanders and van Veen 2011).

In Sumatra, lowland rainforest conversion to smallholder monocultures of rubber and oil palm drives biodiversity decline across animal and plant taxa (Drescher et al. 2016; Grass et al. 2020), leading to functional decline of arthropod communities and associated changes in food web structure and energy fluxes throughout ecosystems (Barnes et al. 2014; Kreider et al. 2021; Pollierer et al. 2023; Potapov et al. 2019, 2024). Nazarreta et al. (2020) showed that monocultures of rubber and oil palm in Sumatra support less than half of the canopy ant species than rainforest, with densities dropping to less than a quarter. Also, monocultures were characterized by a higher proportion of generalist nesters and feeders than rainforest, as well as a higher proportion of ants with large colonies (> 1000 individuals) than rainforest (Kreider et al. 2021). Simultaneously, cash crop monocultures were numerically dominated by invasive ants (Nazarreta et al. 2020), which also contributed to higher trophic positions of canopy ant communities in rubber and oil palm than in rainforest (Pollierer et al. 2023). In contrast, jungle rubber, an extensively used form of rubber agroforestry still common in Sumatra (Gouyon, de Foresta, and Levang 1993), was similar to rainforest in community responses across plant and arthropod taxa (Grass et al. 2020; Potapov et al. 2024; Rembold et al. 2017).

To further our understanding of how tropical land‐use change affects the trophic ecology of canopy ant communities, we here studied trophic positions and community change in acrobat ants from the genus *Crematogaster* lund (1831). This ant genus contributed almost half of the specimens and more than 30 species and morphospecies to the collection of 130,607 worker ants from 226 morphospecies collected in 2013 and 2014 via canopy fogging in the framework of the EFForTS project in lowland Sumatra (Drescher et al. 2016, details see below). *Crematogaster* thus drove much of the observed losses in abundance, diversity, and life‐history traits of the arboreal ant fauna after rainforest conversion to cash crop systems in that area (Kreider et al. 2021; Nazarreta et al. 2020). Similarly, *Crematogaster* contributed much to the biomass of Hymenoptera in those canopy fogging collections, substantially impacting energy fluxes in the canopy food web (Pollierer et al. 2023), and the energy reallocation from green to brown food webs (Potapov et al. 2024) after rainforest transformation to cash crop agriculture in lowland Sumatra. While direct observations and earlier stable isotope analyses suggest that *Crematogaster* is largely omnivorous and generalistic by feeding mainly on honeydew but also obtaining protein through predation (Blüthgen, Gebauer, and Fiedler 2003; Davidson et al. 2003; Longino 2003), much of the trophic ecology of these ecologically highly relevant ants remains unknown.

To explore the trophic ecology of *Crematogaster* and its potential changes with rainforest transformation, we compared the stable isotopic composition of *Crematogaster* ants in rainforest to those in monoculture cash crops. Specifically, we use stable isotopes of carbon and nitrogen in bulk ant tissue along a land‐use gradient from rainforest to monocultures of rubber and oil palm, with plant‐normalized Δ^13^C values as proxy for basal resource use and Δ^15^N values as indicator for trophic position in the food web. In contrast to previous studies focusing on trophic structure of tropical ant assemblages (e.g., Davidson et al. 2003; Pfeiffer, Mezger, and Dyckmans 2014), we adjusted values of Δ^13^C and Δ^15^N to the species‐abundances of more than 12,000 worker ants from 14 Linnaean species. We also explore density, diversity and community composition of *Crematogaster* in these systems, and discuss the connection between community change and trophic ecology of *Crematogaster* in the context of tropical land‐use change.

## 2 Materials and Methods

### 2.1 Study Site

Jambi Province in central Sumatra is one of the hotspots of cash crop agriculture in Indonesia. Smallholder‐dominated mosaics (each about 2–10 ha in size) of forest remnants, “jungle rubber”, and monoculture plantations of rubber and oil palm make up a large part of the lowlands of Jambi Province today (Clough et al. 2016). In 2021, 659,688 ha of rubber and 530,721 ha of oil palm were grown in Jambi Province (BPS 2022), with smallholders cultivating virtually the entire rubber area and about half of the oil palm area (BPS 2023). The vast majority of the remaining forest in Jambi Province is located in mountainous areas, while in the lowlands, only an estimated 4% of rainforest remains (Drescher et al. 2016). “Jungle rubber”, an apparent compromise between productivity on one hand and conservation on the other, is the result of planting rubber into semi‐natural forest regrowth after abandoning rice cultivation in large parts of Jambi at the onset of the 20th century (Gouyon, de Foresta, and Levang 1993). However, this agricultural system is increasingly replaced by rubber and oil palm monocultures, which are much more profitable (Drescher et al. 2016; Grass et al. 2020). Jambi's climate is tropical humid with two main rainy peaks in March and December and a drier season between July and August (Drescher et al. 2016).

### 2.2 Sampling

Acrobat ants (*Crematogaster* spp.) individuals used in this study were sampled in 2017 as part of a canopy fogging campaign in plots of the EFForTS research project (Drescher et al. 2016), and thus 4 years after the sampling campaigns which form the basis of previous publications which also include *Crematogaster* from Sumatra (Kreider et al. 2021; Nazarreta et al. 2020). The canopy fogging campaign targeted all arboreal arthropods from four land‐use systems: (1) lowland rainforest, (2) jungle rubber, and smallholder monoculture plantations of (3) rubber and (4) oil palm. Samples were taken from eight permanent research plots per land‐use system, each plot measuring 50 × 50 m, which were arranged in two clusters such that each plot cluster contained four plots of each land‐use system (Figure S1; Drescher et al. 2016).

*Crematogaster* worker ants used in this study were collected between 03 June and 28 August 2017 from three locations (“subplots”) per each of the 32 research plots, resulting in 96 subplot samples. The three subplots per plot were arranged in a triangle around the plot centre, with canopy gaps and fallen trees being avoided. In each of the subplots, 50 mL DECIS 25 EC (Bayer Crop Science, 25 g/L deltamethrine) mixed in four liters of petroleum oil (“white oil”) were applied to the canopy using a Swingfog SN50 (Swingtec GmBH, Germany). Underneath the canopy in each subplot, eight conical collection traps measuring 1 m ⨯ 1 m were placed (Figure S2). Each trap was fitted with a 250 mL polyethylene bottle filled with 100 mL of 96% ethanol. Two hours after application of the insecticide, the eight PE flasks of each subplot were removed and combined into a single sample. More details on the canopy fogging methods can be found in Pollierer et al. (2023).

### 2.3 Species Identification

First, the mixed canopy arthropods were sorted to insect orders, with the ants (Formicidae) separated from the other insects of the order Hymenoptera. About 33,000 ants were then further sorted to 50 genera (unpublished data by Janotta & Drescher, Desriana & Buchori), based on identification keys to genera in “A Guide to the Ants of Jambi” (Nazarreta et al. 2021) and online sources (AntWeb 2023; AntWiki 2023). In total, 12,185 worker *Crematogaster* ants were encountered, of which 12,125 specimens were identified to 14 Linnaean species and 60 specimens to 12 morphospecies, using genus‐specific identification keys (Blaimer 2012; Hosoichi and Ogata 2009), online sources (AntWeb 2023; AntWiki 2023) and specimens from an earlier collection (Nazarreta et al. 2020, 2021). Only specimens assigned Linnaean species were used in the present study, as they cover more than 99.5% of the abundance in the data, and unidentified morphospecies would increase the importance of very rare species in a diversity analysis. Species‐abundance data was pooled across the three subplots per plot.

### 2.4 Stable Isotope Analysis

Bulk stable isotope analysis was conducted using the pooled “functional community” of *Crematogaster* in each plot. For each plot, the functional community was defined as consisting of every species that cumulatively contributed to at least 90% of the overall *Crematogaster* abundance in each plot, starting with the most abundant species (see Table S1: contribution of species to plot level stable isotope measurements). Up to five individuals of each of the species selected this way were individually subjected to stable isotope measurements. To calibrate stable isotopes to the potential basal resource of the local food web, five randomly selected leaves from up to five different tree species per plot were collected at head height near the plot centre (for details see Pollierer et al. 2023). The five leaves per plot were pooled, dried, milled and a sample of 0.5–1 mg was taken from the powder. Stable isotope ratios of carbon (^13^C/^12^C) and nitrogen (^15^N/^14^N) of both the 208 *Crematogaster* samples as well as 32 leaf samples were measured with a coupled system of an elemental analyzer and a mass spectrometer (Reineking, Langel, and Schikowski 1993). Atmospheric N was used as standard for ^15^N, Vienna Peedee Belemnite (VPDB) for ^13^C and acetanilide (C_8_H_9_NO) as internal standard. Δ^13^C and Δ^15^N values represent differences between ẟ^13^C and ẟ^15^N of *Crematogaster* to ẟ^13^C and ẟ^15^N of leaf material in each plot, i.e., are calibrated values to the potential basal resource of each local food web. Averages of both Δ^13^C and Δ^15^N were first calculated per species per plot and then as averages per plot. The averages were used to prepare abundance‐weighted biplots as well as to calculate abundance weighted stable isotope metrics per plot. Three plots of oil palm and one plot of rubber were excluded from the calculation of stable isotope metrics due to insufficient data (see Table S1, annotations in R script).

### 2.5 Statistical Analysis

All statistical analyses were performed in R version 4.4.1 (R Core Team 2022) and visualized using ggplot2 (Wickham 2016). Diagnostic plots of standard deviation against mean Δ^13^C and Δ^15^N showed that data were normally distributed. Univariate stable isotope metrics ‘Average’, ‘Maximum’, ‘Minimum’ and ‘Range’ were calculated across abundance weighted values per species per plot as in Krause et al. (2021) based on Cucherousset and Villéger (2015). For both Δ^13^C and Δ^15^N, response variables ‘Average’, ‘Maximum’, ‘Minimum’ and ‘Range’ were used as fixed factors in linear models (lm) with R packages ‘nlme’ (Pinheiro et al. 2023) and ‘mass’ (Venables and Ripley 2002), except for ‘Range’ of Δ^15^N which was analyzed with glm with Gaussian family and log link function; predictor variable was ‘land use’ for all response variables.

Relative abundances and community overlaps of *Crematogaster* were visualized using rank abundance curves (RankAbund; Hartke 2020) and Venn diagrams as well as the species pool calculated (vegan; Oksanen et al. 2019). To calculate rarity, species weights were first taken as the inverse of the number of samples in which they occurred, ranging from “1” for the rarest species which occurred in only one plot (1/1 = 1) and “0.03125” for the most common species which occurred in all plots (1/32 = 0.03125). Rarity was then calculated per plot as the sum of the weights for the individual species found in that plot. The response variables abundance, richness and rarity were compared between land uses using generalized linear models (glm) with negative binomial family (log link function; abundance), Gaussian family (identity link function; richness and rarity). Full models including the interaction between land use and landscape (cluster) were simplified to the most parsimonious model based on AICc (Bartoń 2024). ANOVA was used to test for significant deviation from the null model, after checking model fit with DHARMa (Hartig 2022). Compositional dissimilarity of *Crematogaster* communities among land uses was tested with multivariate glm (Wang et al. 2021), and community composition visualized using a latent variable model in boral (bayesian ordination and regression analysis; Hui 2016) and ggboral (Bedward 2020).

## 3 Results

**FIGURE 1:**
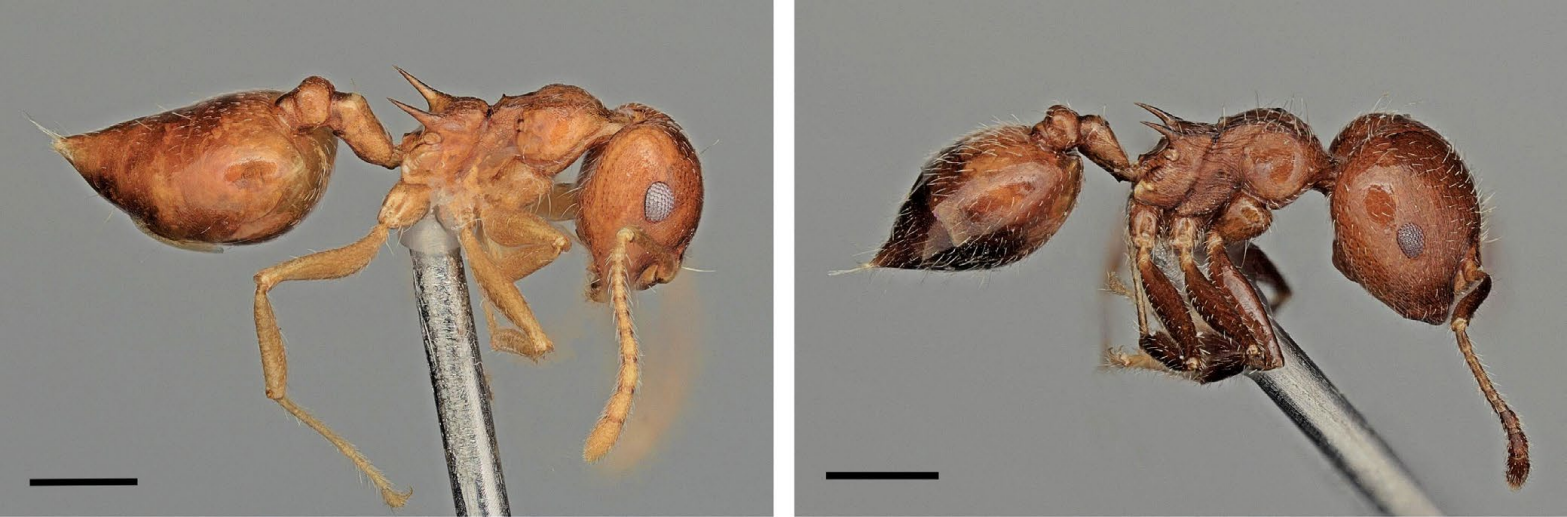
Two acrobat ant species, *Crematogaster coriaria* (left) and *C. fraxatrix* (right), contributed more than 60% of the total abundance in canopy fogging samples from rainforest, jungle rubber, and smallholder plantations of rubber and oil palm in Jambi, Sumatra, Indonesia.

In the dry season of 2017, 12,185 *Crematogaster* worker ants were collected, with more than 99.5% (*N* = 12,125) identified to 14 Linnaean species. Cumulative abundance of *Crematogaster* species and individuals was highest in rainforest (13 species and 6819 individuals) and jungle rubber (10 species and 4561 individuals). Significantly fewer individuals were sampled from rubber (4 species and 302 individuals) and oil palm (4 species and 443 individuals). Among the 14 Linnaean species, *C. coriaria* and *C. fraxatrix* accounted for more than 60% of overall individuals (*N* = 3905 and *N* = 3377, respectively; Figure 1). In contrast, six species, representing approximately 40% of the species pool were represented by fewer than 50 individuals each. Except for *C. ferrarii* , which was found exclusively in oil palm sites, these rare species were collected solely from rainforest and jungle rubber. Despite an overall decline in *Crematogaster* abundance across the land‐use gradient, two species, *Crematogaster rogenhoferi* and *C. treubi* , occasionally occurred in high abundance in plantations.

### 3.1 Trophic Structure

**FIGURE 2:**
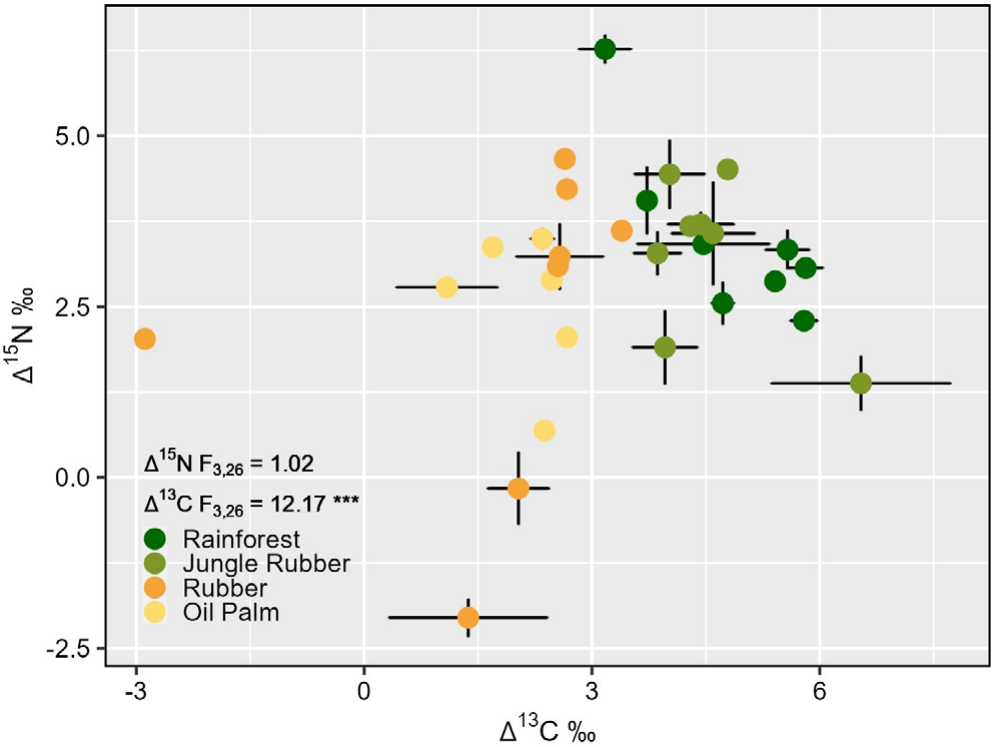
Biplot of leaf calibrated bulk stable isotopes (BSI) Δ^13^C and Δ^15^N of *Crematogaster* communities from four land‐use systems in Jambi, Sumatra, Indonesia. Dots represents the abundance‐weighted means ± SD (if applicable) of individual plots in a research design of eight plots per land‐use system, with two plots in oil palm discarded (no *Crematogaster* in one plot, insufficient stable isotope data in another). *F*‐test statistics correspond to the influence of land use on average Δ^13^C and Δ^15^N.

**FIGURE 3:**
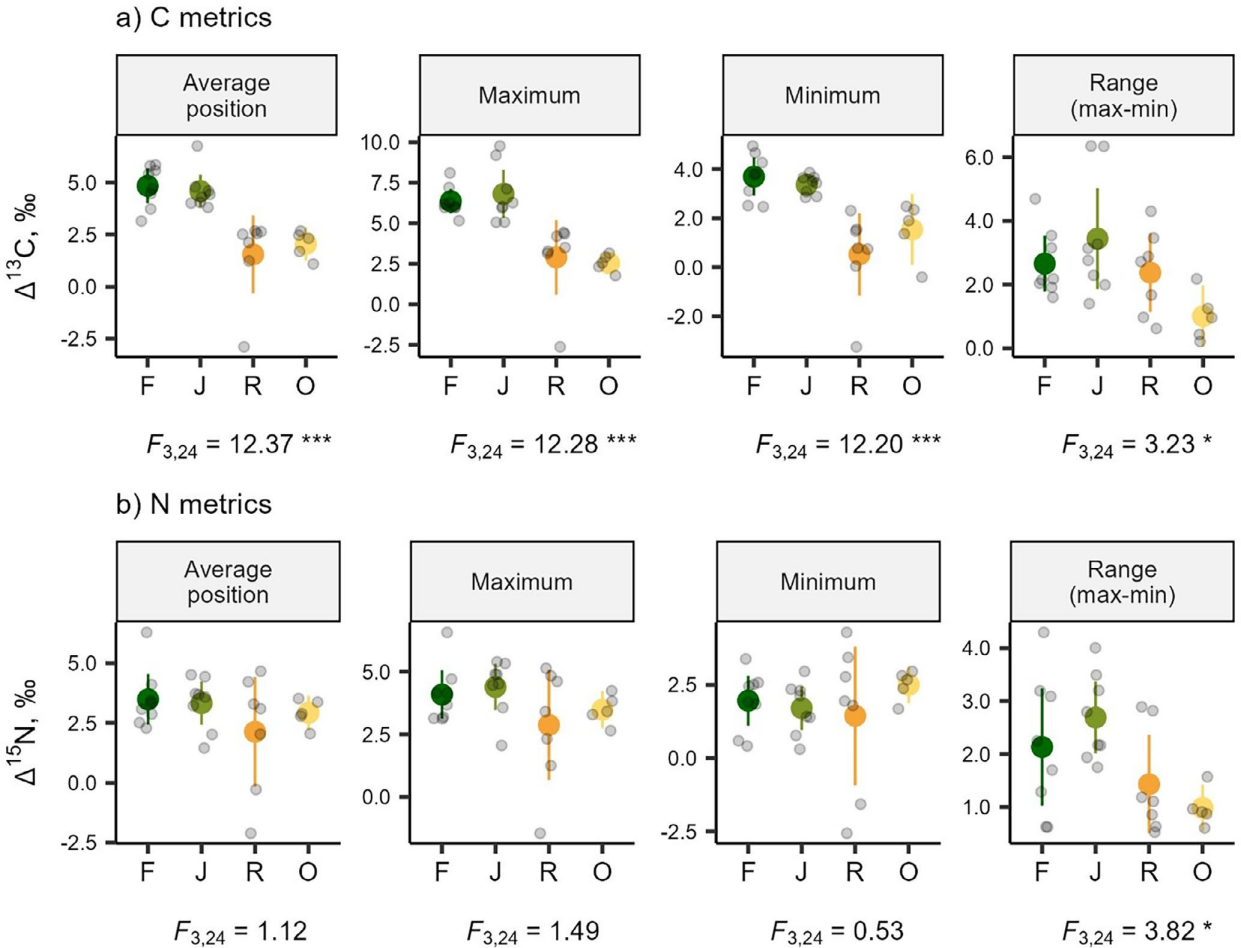
Abundance‐weighted Average, Maximum, Minimum and Range of Δ^13^C (a) and Δ^15^N (b) of *Crematogaster* communities from four land‐use systems in Jambi, Sumatra, Indonesia. Gray dots represent data from individual plots, while colored, enlarged dots represent means ± sd across plots (dark green = rainforest, ‘F’; green = jungle rubber ‘J’; orange = rubber ‘R’; yellow = oil palm ‘O’). Test statistics are GLM followed by ANOVA. *P < 0.05; **P < 0.01; ***P < 0.001.

*Crematogaster* communities from the more natural systems rainforest and jungle rubber were well separated from the monoculture plantations of rubber and oil palm in a biplot of Δ^13^C and Δ^15^N values per plot (Figure 2). Contrasts in the average stable isotopic positions of *Crematogaster* communities between land‐use systems were very highly pronounced for Δ^13^C (*F*_3,26_ = 12.17, *p* < 0.001) but not in Δ^15^N (*F*_3,26_ = 1.02, *p* = 0.4). Land use significantly influenced the Δ^13^C metrics of *Crematogaster* (Figure 3a; *F*_3,24_ = 12.37, *p* < 0.001 for ‘Average’; *F*_3,24_ = 12.28, *p* < 0.001 for ‘Maximum’; *F*_3,24_ = 12.2, *p* < 0.001 for ‘Minimum’; *F*_3,24_ = 3.23, *p* = 0.04 for ‘Range’). *Crematogaster* from rainforest and jungle rubber were more enriched in ^13^C compared to leaf material, as indicated by maximum Δ^13^C values of 6.35‰ ± 0.9‰ and 6.81‰ ± 1.78‰ (means ± SD), respectively. Moreover, ants from rainforest and jungle rubber maintained minimum Δ^13^C values greater than 2‰, while ants from rubber and oil palm had lower minimum Δ^13^C values (0.52‰ ± 1.8‰ and 1.5‰ ± 1.17‰, respectively). Maximum, minimum and average positions of *Crematogaster* Δ^15^N values did not differ significantly among the land‐use systems. However, the range of Δ^15^N values differed between land‐use systems (*F*_3,24_ = 3.82, *p* = 0.022), with higher within‐plot ranges in rainforest and jungle rubber compared to oil palm and rubber plantations (Figure 3b).

### 3.2 Diversity and Community Structure

**FIGURE 4:**
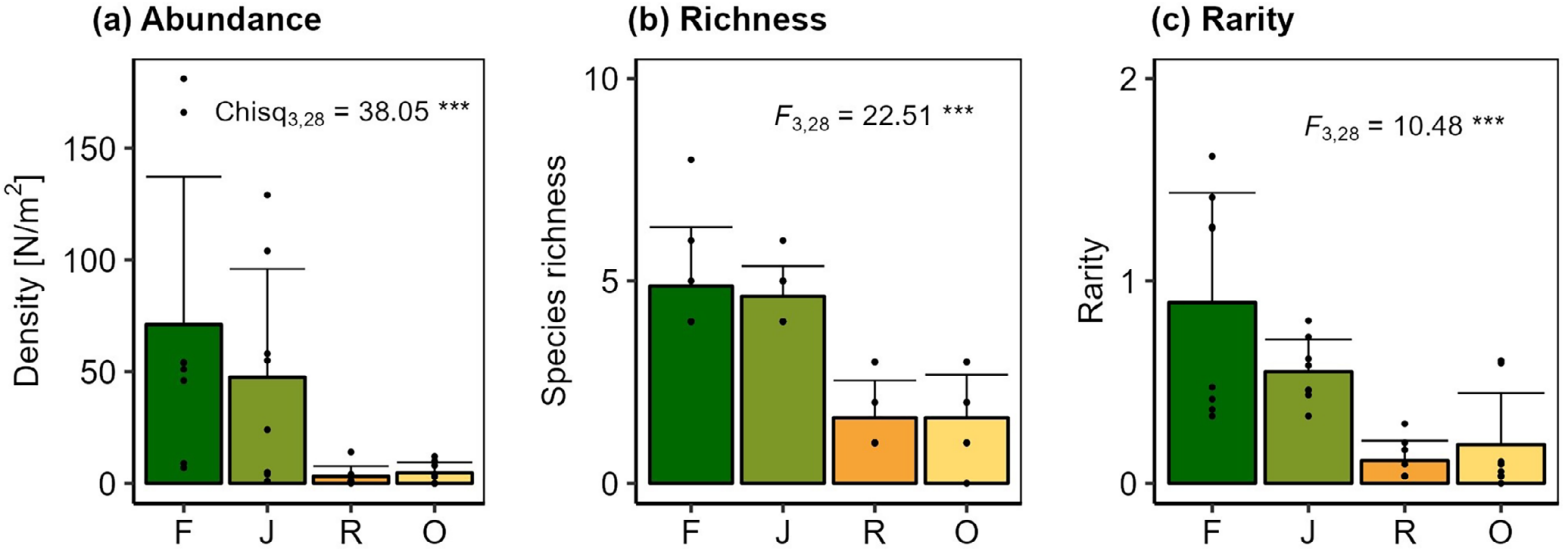
Average and standard deviation of *Crematogaster* (a) abundance (N/m^2^) (b) species richness and (c) rarity in four major land‐use systems in Jambi, Sumatra, Indonesia (dark green = rainforest, ‘F’; green = jungle rubber ‘J’; orange = rubber ‘R’; yellow = oil palm ‘O’). Dots represent data from individual plots.

**FIGURE 5:**
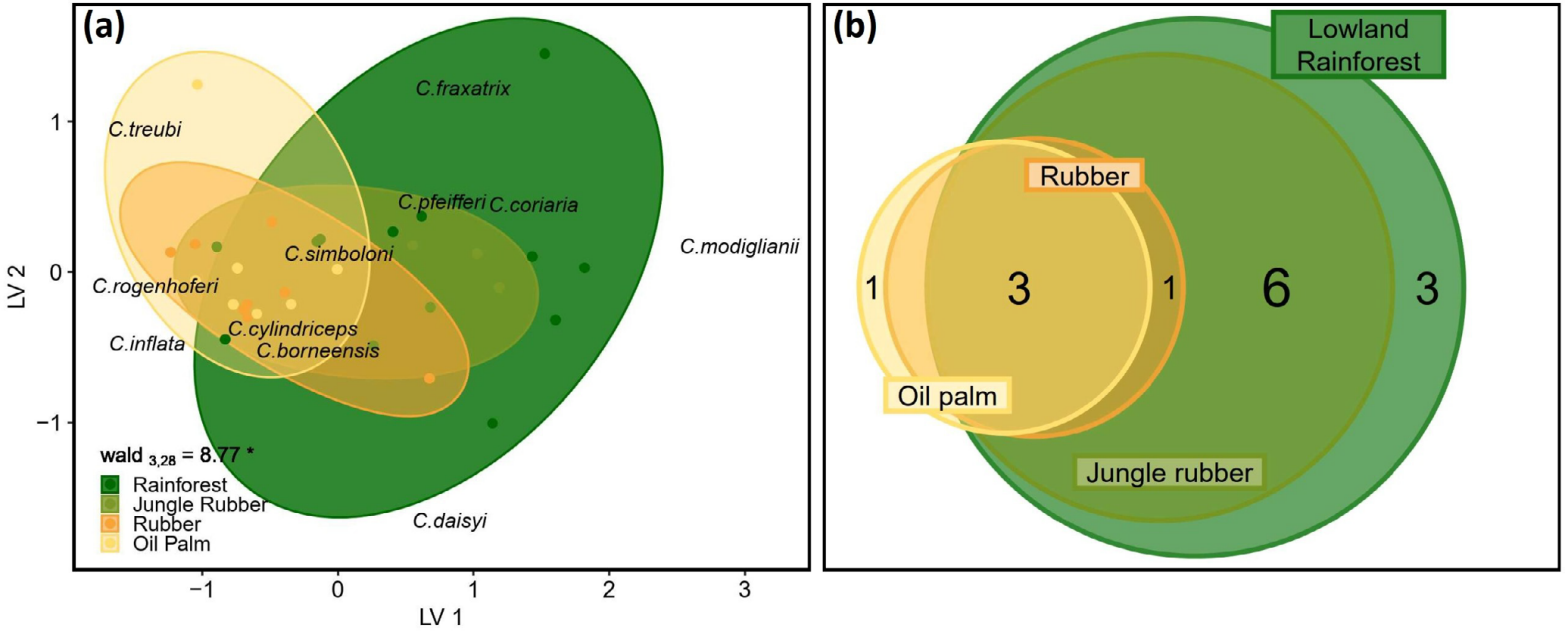
(a) Latent variable biplot of *Crematogaster* communities in four major land‐use systems in Jambi, Sumatra, Indonesia. Dots represent data from individual plots, species are positioned in accordance to their habitat affiliation, and wald test statistics indicate significant influence of ‘land use’ on community composition of *Crematogaster* after multivariate glm. (b) Venn diagram of *Crematogaster* communities overlap across four land‐use systems in Jambi, Sumatra, Indonesia, with circle size corresponding to the number of species found exclusively here.

The average abundance of *Crematogaster* was significantly influenced by land use (*Χ* ^2^ = 38.05, *p* < 0.001). Specifically, *Crematogaster* abundance in rainforest (71.0 ± 66.2 ind./m^2^, means ± sd) was more than 15 times higher than in oil palm (4.6 ± 4.8 ind./m^2^) and more than 22 times higher than in rubber (3.1 ± 4.6 ind./m^2^). While *Crematogaster* abundance in jungle rubber (47.5 ± 48.4 ind./m^2^) was markedly lower than that in rainforest, is was still much higher than in the monoculture plantations (Figure 4a). Species richness of *Crematogaster* likewise was significantly influenced by land use (*F*_3,28_ = 22.5, *p* < 0.001), with average species richness in rainforest (4.9 ± 1.5) and jungle rubber (4.6 ± 0.7) approximately three times higher than in rubber (1.6 ± 0.9) or oil palm plantations (1.6 ± 1.0) (Figure 4b). *Crematogaster* rarity was significantly influenced by land use as well (*F*_3,28_ = 10.48, *p* < 0.001), with highest average rarity in rainforest (0.90 ± 0.54), intermediate rarity in jungle rubber (0.55 ± 0.16), and lowest rarity in rubber and oil palm (0.11 ± 0.09 and 0.19 ± 0.26; Figure 4c). Ranked cumulative abundances and species richness of *Crematogaster* were much higher in rainforest and jungle rubber than in rubber and oil palm (Figure S5). Species accumulation curves indicate sufficient coverage for rubber and oil palm, but suggest considerable undersampling in rainforest and jungle rubber (Figure S6). Chao's estimated species pool in rainforest was *S* = 33.4 ± 27.3, (observed *S* = 12), *S* = 12.0 ± 3.0 in jungle rubber (observed *S* = 10), *S* = 4.4 ± 1.2 in rubber (observed *S* = 4) and *S* = 3.0 ± < 0.1 in oil palm (observed *S* = 3). Community composition of *Crematogaster* ants was significantly influenced by land use (wald = 8.77, *p* = 0.012; Figure 5a), and communities in rubber and oil palm were almost perfect subsamples of those in rainforest and jungle rubber (Figure 5b).

## 4 Discussion

Here, we examined the consequences of tropical rainforest transformation into rubber and oil palm smallholder plantations on the trophic niche and community structure of acrobat ants of the genus *Crematogaster*. Bulk stable isotopes of ^13^C suggest a trophic shift of *Crematogaster* towards plant‐based resources in the monocultures, but that pattern may be driven by the “canopy effect”, i.e. differential accumulation of ^13^C in leaves from different heights. On the other hand, ^15^N bulk stable isotopes indicate significantly reduced feeding variability of *Crematogaster* in rubber and oil palm monocultures. These trophic changes within the *Crematogaster* communities coincide with substantial losses in abundance, species richness and rarity, accompanied by altered community composition. In an ant genus as abundant as *Crematogaster*, altered feeding strategies and a heavily changed community likely affects ecosystem services provided by this taxon through lowered redundancy and stability in cash crop monocultures in Southeast Asia.

### 4.1 Trophic Structure After Land‐Use Change

The conversion of rainforest to monoculture plantations of rubber and oil palm led to shifts in trophic niches, as indicated by abundance‐weighted and leaf‐calibrated isotopic signatures of ^15^N and ^13^C. We weighted single‐individual based isotopic signatures of worker ants from common species by the abundance of those species, replicated per plot. This approach distinguishes our study from the most commonly used methods, which often measure every species in a community without abundance correction (e.g., Davidson et al. 2003; Pfeiffer, Mezger, and Dyckmans 2014). The typical approach may disproportionately represent rare species, particularly those from small colonies, thus giving them a higher relative weight in a sample compared to common, abundant species. The trophic patterns of *Crematogaster* presented here are likely more representative of many ecosystems, where common and abundant species (or those with high biomass) drive ecological patterns rather than rare species (but see Arnoldi, Loreau, and Haegeman 2019; e.g. Winfree et al. 2015).

On average, *Crematogaster* ants showed an enrichment of approximately 2‰–3‰ in ^15^N compared to plant leaves, suggesting a trophic position akin to herbivores, which raises questions about their classification as omnivores (Blüthgen, Gebauer, and Fiedler 2003; Davidson et al. 2003). Since most *Crematogaster* ants are arboreal, they likely heavily rely on honeydew. However, Sagers and Goggin (2007) reported a 1.4‰ enrichment of ^15^N in honeydew compared to the leaves' isotopic signatures. Assuming a mean enrichment of 2.2‰ per trophic level (McCutchan Jr et al. 2003), ants relying on honeydew should have higher enrichment than we observed for *Crematogaster* here. As suggested by Cook and Davidson (2006), bacterial endosymbionts may be responsible for ants appearing “more herbivorous than they actually are” due to nitrogen‐upgrading or recycling, particularly when they rely heavily on trophobioses. This suggests that any occasional predation or scavenging by *Crematogaster* might be challenging to detect through stable isotopes alone, especially if they indeed rely heavily on trophobiosis. In addition, predation or scavenging within *Crematogaster* or the broader canopy food web may be led detectable if prey items have particularly low δ^15^N values due to feeding on algae or lichens, such as arboreal springtails or barklice (Collembola, Psocoptera; Pollierer et al. 2023).

Conversely, compared to leaves, *Crematogaster* ants were less depleted in δ^13^C, particularly in rainforest and jungle rubber, with the increase in δ^13^C values even exceeding that of δ^15^N. High Δ^13^C values of *Crematogaster* ants in rainforest and jungle rubber mirror the general δ^13^C patterns of canopy arthropods in these systems and argue against a purely plant‐based diet (Pollierer et al. 2023). However, this may possibly be attributed to the “canopy effect”, where leaves from lower canopies tend to be more depleted in ^13^C since much of the CO_2_ they assimilate originates from soil respiration. In contrast, leaves from higher canopies predominantly assimilate CO_2_ from the atmosphere (van der Merwe and Medina 1991). Since rainforest and jungle rubber have taller and denser canopies compared to monocultures of rubber and oil palm (Drescher et al. 2016; Kotowska et al. 2015), calibrating *Crematogaster* ants to lower canopy leaves, as done here, may account for some of the observed differences in average and maximum Δ^13^C values. In comparison, uncalibrated bulk stable isotopic values of ẟ^13^C show a somewhat opposite pattern for average, maximum and minimum (but not for range) than calibrated Δ^13^C (Figure S3). A supplementary regression analysis with leaf litter from the same plots (which also includes leaves from high canopy) indicates differences of ~2‰ to 3‰ between δ^13^C of leaf litter and lower canopy leaves in jungle rubber and rainforest, respectively, suggesting a contribution of the canopy effect (Figure S4). However, lower Δ^13^C values of *Crematogaster* in natural systems compared to plantations may also reflect a closer association with plant‐based resources, or a higher degree of predation or scavenging of algae‐microbivorous prey in the latter (Pollierer et al. 2023). Interestingly, variation in *Crematogaster* trophic niches between plots was highest in rubber plantations in the Harapan region (Figure S7), suggesting that a landscape or regional contexts of transformation also play a role in shaping the trophic structure of *Crematogaster* communities. Generally, however, within‐plot variation of stable isotope signatures–i.e., the range of both Δ^13^C and Δ^15^N values–was lower in rubber and oil palm plantations than in more natural ecosystems. This might indicate reduced resource availability and, therefore, a reduced trophic niche size, but it could also result from reduced species diversity in rubber and oil palm plantations which are dominated by *C. rogenhoferi* and *C. treubi* . However, at least in rubber, the high between‐plot variation argues against the latter. Nevertheless, reduced trophic diversity implies reductions or losses in functional diversity, potentially compromising ecosystem multifunctionality (Soliveres et al. 2016).

### 4.2 Diversity and Community Composition After Land‐Use Change

In conjunction with trophic changes within the community, *Crematogaster* abundance was highest in rainforest and jungle rubber and lowest in rubber and oil palm plantations. Our results reflect previous studies reporting higher canopy arthropod abundances in rainforest compared to monoculture plantations (Azhar et al. 2022; Kasmiatun Hartke et al. 2023; Mawan et al. 2022; Pollierer et al. 2023; Ramos et al. 2022). Habitat disturbance due to land‐use intensification is known to reduce arthropod population sizes. Factors like pesticide use, fertilization (Kytö, Niemelä, and Larsson 1996) and loss of habitat heterogeneity (Teuscher et al. 2016) may play an essential role. Additionally, higher aboveground temperatures, either resulting from increased canopy openness or driven by climate change, have been shown to reduce arthropod biomass (Aucott 2019; Lister and Garcia 2018). Average *Crematogaster* abundance decreased by over 33% in jungle rubber, by more than 90% in oil palm and up to 95% in rubber compared to rainforest. This is more pronounced than the overall decrease in the ant community (> 75% from rainforest to oil palm and > 80% from rainforest to rubber; Nazarreta et al. 2020), suggesting that some species of *Crematogaster* may be particularly sensitive to land‐use change. However, different sampling techniques may also have contributed to the variation in results between the present study and the one of Nazarreta et al. (2020). For the canopy fogging samples used by Nazarreta et al. (2020), 16 traps of 1 m^2^ were placed below the targeted canopy for each subplot (=48 traps per plot), whereas only half as many traps were set up for sampling in this study (8 traps per subplot, 24 traps per plot). Reduced sampling effort in the 2017 collection used here compared to the 2013 collection by Nazarreta et al. (2020) may lead to not only lower numbers but likely fewer species as well (Gotelli and Colwell 2001). Nevertheless, consistent with Nazarreta et al. (2020), the present study also found *Crematogaster rogenhoferi* and *C. treubi* to be highly abundant in disturbed habitats and omnipresent in all four land‐use systems. Lower *Crematogaster* abundance in rubber compared to oil palm plantations may also be attributed to a decrease in epiphyte abundance in rubber (Böhnert et al. 2016), which is known to correlate with canopy ant abundance (Stuntz et al. 2003). Further, the shedding of leaves by rubber in the dry season may contribute to low canopy arthropod abundance in rubber monoculture plantations. In contrast, there was a relatively small decline in *Crematogaster* abundance in jungle rubber, presumably due to the low land‐use intensity and high proportion of forest tree species (Rembold et al. 2017).

In comparison to rainforest, species richness was slightly different in jungle rubber but reduced by more than half in rubber and oil palm. Notably, differences in species richness were substantial between the more natural systems (rainforest and jungle rubber) and the high‐intensity land‐use systems (rubber and oil palm). Our finding supports previous studies that compared ant diversity in rainforests with oil palm sites in Southeast Asia (Brühl and Eltz 2010; Fayle et al. 2010; Pfeiffer, Tuck, and Lay 2008). As mentioned above, this is likely due to the decrease in plant diversity from rainforest to rubber and oil palm, which leads to a reduction in ecological niches, thereby negatively affecting arthropod population sizes and species richness. For ants in particular, the availability of nesting sites is a limiting factor, driving segregation and mosaic like patterns in oil palm plantations but not in rainforest, where weaker nest‐site limitation and high horizontal and vertical structural complexity may facilitate more co‐occurrence between arboreal ant taxa including *Crematogaster* (Fayle, Turner, and Foster 2013; Yusah et al. 2018). Importantly, decreases in species number were not accompanied by pronounced shifts in community composition, but were mainly characterized by losses from the overall species pool as communities in plantations were subsets of rainforest communities. This suggests that *Crematogaster* communities cannot adapt to the changing habitat conditions in plantation systems, presumably leading to pronounced functional losses. As an exception, *Crematogaster ferrarii* was only found in oil palm plantations and dominated *Crematogaster* communities there. Little is known about *C. ferarrii*'s ecology, but it has been collected from “waste woodland secondary forest” and old growth mangroves (Wang et al. 2022), suggesting it may be a generalist capable of coping with disturbances, much like tramps or invasive ants. The increased dominance of those generalist ants in monocultures (Nazarreta et al. 2020) may further negatively affect *Crematogaster* abundance and richness by aggressive displacing them at food or other resources (Drescher, Feldhaar, and Blüthgen 2011).

## 5 Conclusion

The present study adds to the understanding of the effects of tropical land‐use change on the numerous but understudied acrobat ants (*Crematogaster*) in Southeast Asia. *Crematogaster* communities in monoculture plantation systems had lower trophic diversity and, in case of rubber plantations, more random community trophic niches compared to those in rainforest. While in part explicable by the ‘canopy effect’, the observed shifts also indicate a closer association with plant or algae‐based resources. The density and diversity of *Crematogaster* were heavily reduced along the studied land‐use gradient, and *Crematogaster* community composition in plantation systems formed subsets of the rainforest community. These observed patterns suggest that functional losses of acrobat ants after rainforest conversion to cash crop plantations may be even more pronounced than in the overall ant community. Lastly, our study highlights the importance of investigating individual taxa to understand the intricate and complex consequences of tropical land‐use change on agricultural ecosystem functions and services.

## Author Contributions

**Jessica Ehlers:** data curation (equal), formal analysis (equal), investigation (equal), methodology (equal), writing – original draft (equal), writing – review and editing (equal). **Tamara R. Hartke:** data curation (equal), formal analysis (equal), investigation (equal), supervision (equal), writing – original draft (equal), writing – review and editing (equal). **Noah Janotta:** data curation (supporting), formal analysis (supporting), writing – review and editing (supporting). **Amanda Mawan:** investigation (supporting). **Rizky Nazarreta:** data curation (supporting), investigation (supporting), writing – review and editing (supporting). **Rizky Desriana:** data curation (supporting), investigation (supporting), methodology (supporting). **Purnama Hidayat:** investigation (supporting), project administration (supporting), resources (supporting), supervision (supporting), writing – review and editing (supporting). **Damayanti Buchori:** investigation (supporting), project administration (supporting), resources (supporting), supervision (supporting), writing – review and editing (supporting). **Stefan Scheu:** conceptualization (equal), funding acquisition (lead), investigation (equal), project administration (lead), resources (lead), supervision (supporting), writing – original draft (supporting), writing – review and editing (supporting). **Melanie M. Pollierer:** conceptualization (equal), data curation (equal), formal analysis (equal), investigation (equal), methodology (equal), supervision (equal), validation (equal), visualization (equal), writing – original draft (equal), writing – review and editing (equal). **Jochen Drescher:** conceptualization (equal), data curation (equal), formal analysis (equal), investigation (equal), methodology (equal), project administration (equal), supervision (equal), validation (equal), visualization (equal), writing – original draft (lead), writing – review and editing (lead).

## Conflicts of Interest

The authors declare no conflicts of interest.

## Supporting information


Data S1.

## Data Availability

All raw data, metadata and analysis code is permanently available under https://doi.org/10.25625/DSSI8M on the GöttingenResearchOnline data sharing platform (https://data.goettingen‐research‐online.de).
