# Supplementary material for: Trophic Change and Community Decline in Acrobat Ants After Rainforest Conversion to Cash Crops
Source: Ecol Evol. 2024 Dec 23;14(12):e70694. doi: 10.1002/ece3.70694 (PMC11664207; doi:10.1002/ece3.70694)
Supplement: Supplementary file 1 — Data S1. [file ECE3-14-e70694-s001.docx]

**SUPPLEMENTARY FIGURES:**

Figure S1: Study site, replication design

Figure S2: Canopy fogging method details

Figure S3: Average, maximum, minimum and range of uncalibrated ẟ13C and ẟ15N

Figure S3: Regressions of ẟ13C and ẟ15N from leaves versus leaf litter

Figure S4: Ranked abundances of *Crematogaster* community

Figure S5: Species accumulation curves of *Crematogaster* community

Figure S6: Rarefied, Randomized species richness of *Crematogaster* community

Figure S7: Abundance weighted average Δ13C and Δ15N per *Crematogaster* species per plot

**SUPPLEMENTARY TABLES:**Table S1: *Crematogaster* species used in bulk stable isotope analysis (BSIA), and the percentage of the *Crematogaster* abundance they represent per plot and land-use system


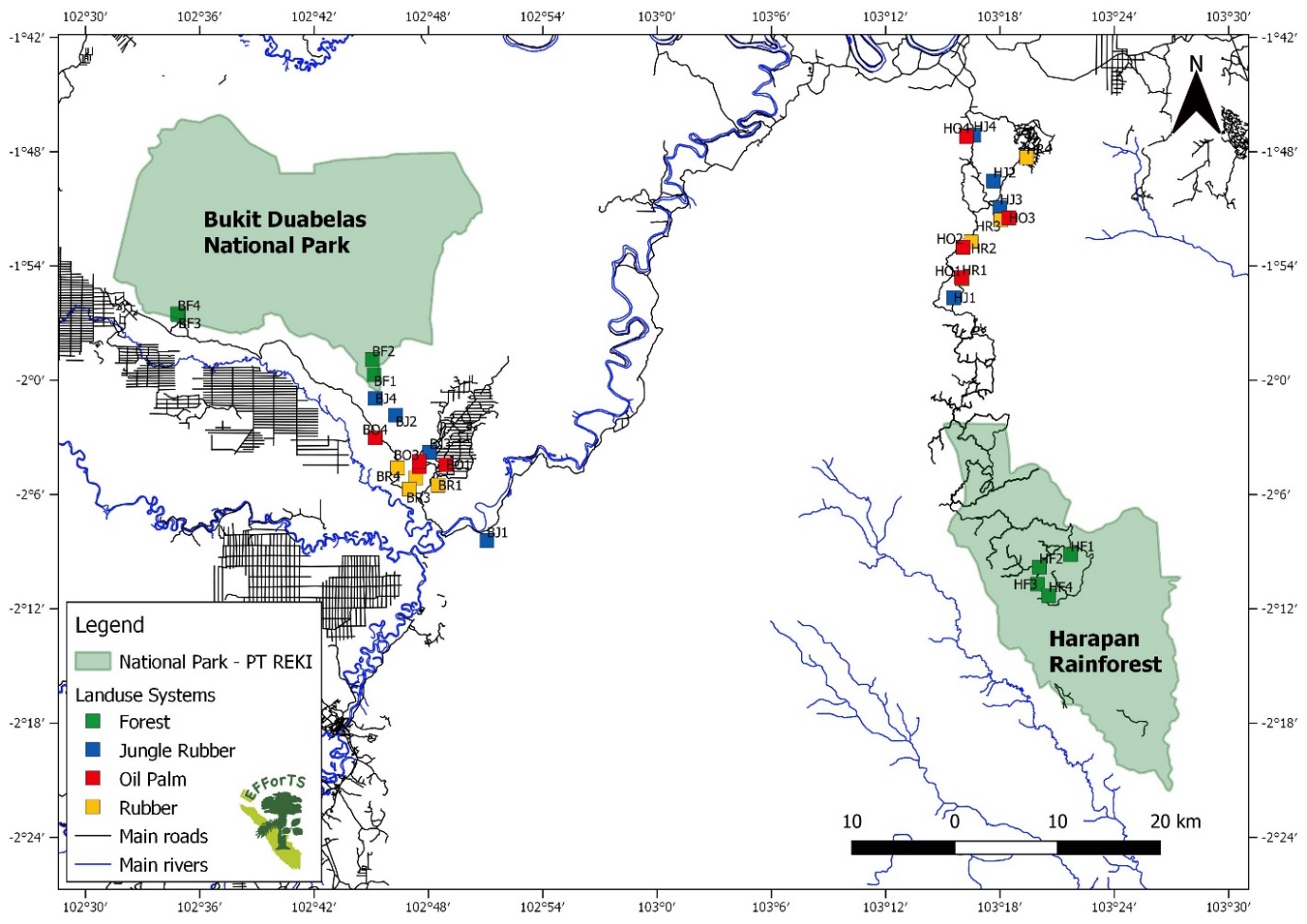


**FIGURE S1.** Replication design and location of 32 study plots in Jambi Province, Sumatra, Indonesia. Plots were arranged into two clusters or “landscapes”, i.e. “Bukit Duabelas” and “Harapan”. Land uses under investigation are color coded: green = rainforest; blue = jungle rubber; yellow = rubber; red = oil palm) (from Ramos & al. 2022).


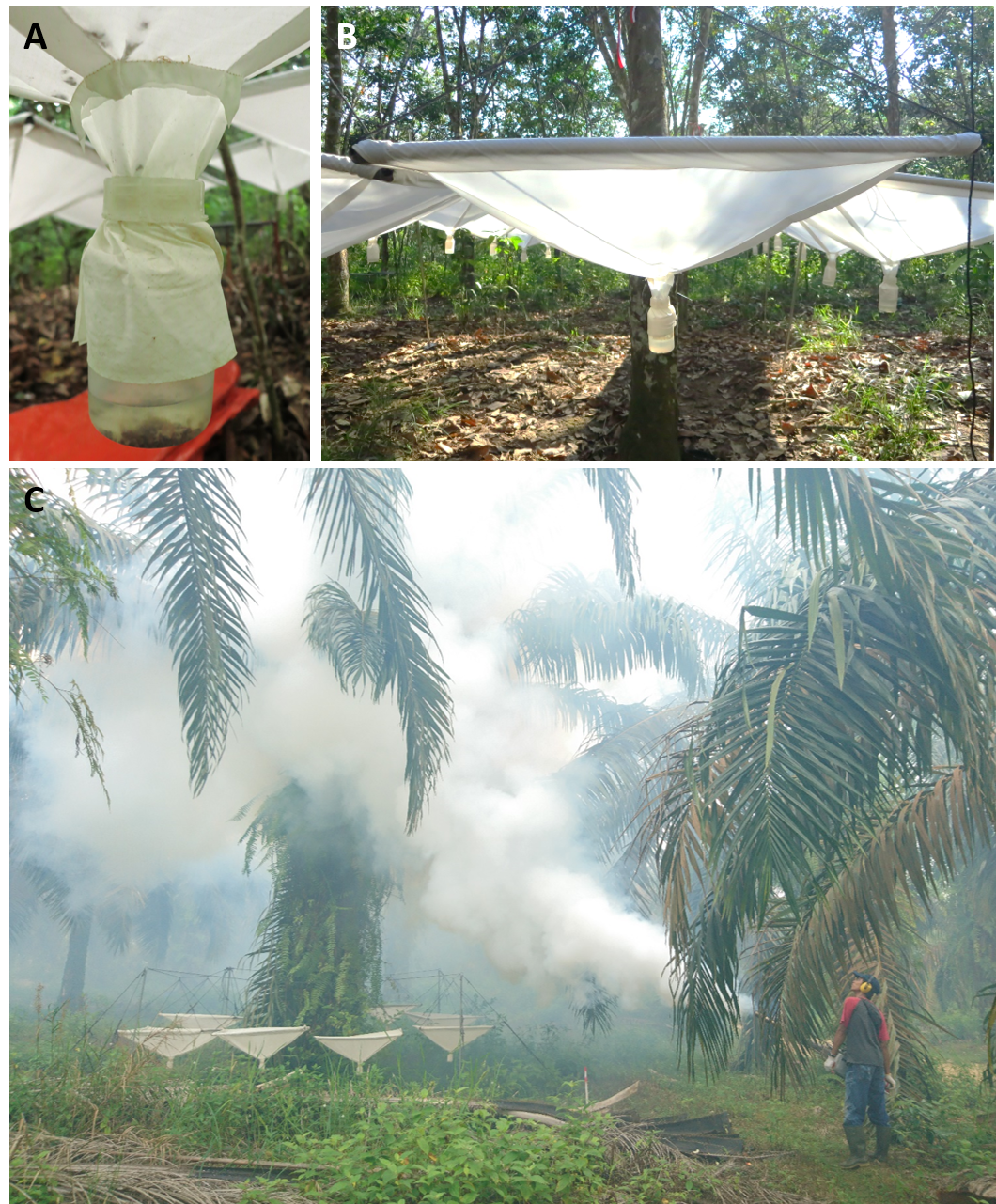


**FIGURE S2 (above).** Canopy fogging setup: (A) 250 ml collection vials containing 100 ml ethanol were attached to (B) homemade collection traps made from balloon cloth, each 1 m × 1 m, hung from ropes between height adjustable tent poles. (C) Eight of the collection traps were used to collect stunned or killed canopy arthropods in each of three subplot replicates per research plot. To each subplot replicate 50 ml DECIS 25 dissolved in 4 l white oil was applied using the SwingFog SN50 fogger (see Methods).

**TABLE S1 (below). I**ndividual and combined contribution of *Crematogaster* species selected for bulk stable isotope analysis to abundance per plot (in %) and land use system (in %).

| **Plot** | ***Crematogaster* spp. selected for BSIA per plot (alphabetically) and their contribution to overall *Crematogaster* abundance per plot (in %)** | | **Cumul. contrib. per plot [%]** | **land-use system** | **Average contribution per land use system** |
| --- | --- | --- | --- | --- | --- |
| **BF1** | *C. coriaria* (95.7), *C. fraxatrix* (2.1),  *C. rogenhoferi* (0.9), *C. treubi* (0.5) | | 99,2 | Lowland Rainforest | 96,4 % |
| **BF2** | *C. coriaria* (72.6), *C. fraxatrix* (27.2),  *C. rogenhoferi* (0.1) | | 99,9 |  |  |
| **BF3** | *C. fraxatrix* (93.9), *C. rogenhoferi* (1.8) | | 95,7 |  |  |
| **BF4** | *C. rogenhoferi* (58), *C. sewardi* (32.1) | | 90,1 |  |  |
| **HF1** | *C. daisyi* (91.7), *C. fraxatrix* (0.8),  *C. rogenhoferi* (0.6) | | 93,1 |  |  |
| **HF2** | *C. fraxatrix* (66.1), *C. modiglianii* (31),  *C. rogenhoferi* (1.2), *C. simboloni* (0.2) | | 98,5 |  |  |
| **HF3** | *C. fraxatrix* (2.4), *C. modiglianii* (94.4),  *C. rogenhoferi* (1.4), *C. treubi* (0.2) | | 98,4 |  |  |
| **HF4** | *C. coriaria* (96.6) | | 96,6 |  |  |
| **BJ3** | *C. rogenhoferi* (6.1), *C. treubi* (89.8) | | 95,9 | Jungle Rubber | 92 % |
| **BJ4** | *C. borneensis* (16.7), *C. coriaria* (43.7),  *C. rogenhoferi* (9.4), *C. sewardi* (25.2), *C. treubi* (2.4) | | 97,4 |  |  |
| **BJ5** | *C. fraxatrix* (68), *C. rogenhoferi* (28.8),  *C. simboloni* (0.1), *C. treubi* (2.6) | | 99,5 |  |  |
| **BJ6** | *C. coriaria* (98.9), *C. rogenhoferi* (0.2), *C. treubi* (0.2) | | 99,3 |  |  |
| **HJ1** | *C. fraxatrix* (97.8), *C. rogenhoferi* (1.6), *C. treubi* (0.5) | | 99,9 |  |  |
| **HJ2** | *C. modiglianii* (45.7), *C. pfeifferi* (30),  *C. rogenhoferi* (14.3), *C. treubi* (1.4) | | 91,4 |  |  |
| **HJ3** | *C. fraxatrix* (93), *C. rogenhoferi* (4.2), *C. simboloni* (1.7) | | 98,9 |  |  |
| **HJ4** | *C. rogenhoferi* (46.7), *C. simboloni* (6.7) | | 53,4 |  |  |
| **BO2** | *C. rogenhoferi* (83.3), *C. simboloni* (2.6) | | 85,9 | Rubber | 94,8 % |
| **BO3** | *C. rogenhoferi* (97.3) | | 97,3 |  |  |
| **BO4** | *C. rogenhoferi* (84.4), *C. treubi* (3.1) | | 87,5 |  |  |
| **BO5** | *C. ferarii* (28.9), *C. rogenhoferi* (68.9) | | 97,8 |  |  |
| **HO2** | *C. treubi* (100) | | 100 |  |  |
| **HO3**^†^ | *C. rogenhoferi* (100), only one BSI data point | | 100 |  |  |
| **HO1**^†^ | NA (BSI data insufficient) | | NA | NA | NA |
| **HO4**^†^ | NA (no *Crematogaster* ants in this plot) | | NA | NA | NA |
| **BR1** | *C. rogenhoferi* (36.9), *C. treubi* (60.7) | | 97,6 | Oil Palm | 99,7 % |
| **BR2, BR3, BR4** | | *C. rogenhoferi* (100) | 100 |  |  |
| **HR1** | *C. borneensis* (100) | | 100 |  |  |
| **HR2** | *C. rogenhoferi* (63.9), *C. treubi* (36.1) | | 100 |  |  |
| **HR3** | *C. fraxatrix* (16.7), *C. rogenhoferi* (63.9), *C. treubi* (25) | | 100 |  |  |
| **HR4**^†^ | *C. rogenhoferi* (100), only one BSI data point | | 100 |  |  |

* In HJ4, only 15 ant individuals were found, 12 of which were kept as reference material. Only three specimen of two species were used for BSIA, leading to low relative contribution scores.

^†^ HO1 and HO4 were excluded from the biplot of leaf calibrated bulk stable isotopes (Fig. 2), and HO1, HO3, HO3 and HR4 were excluded from the calculation of one dimensional BSIA metrics ‘ (Fig, 3 main manuscript).


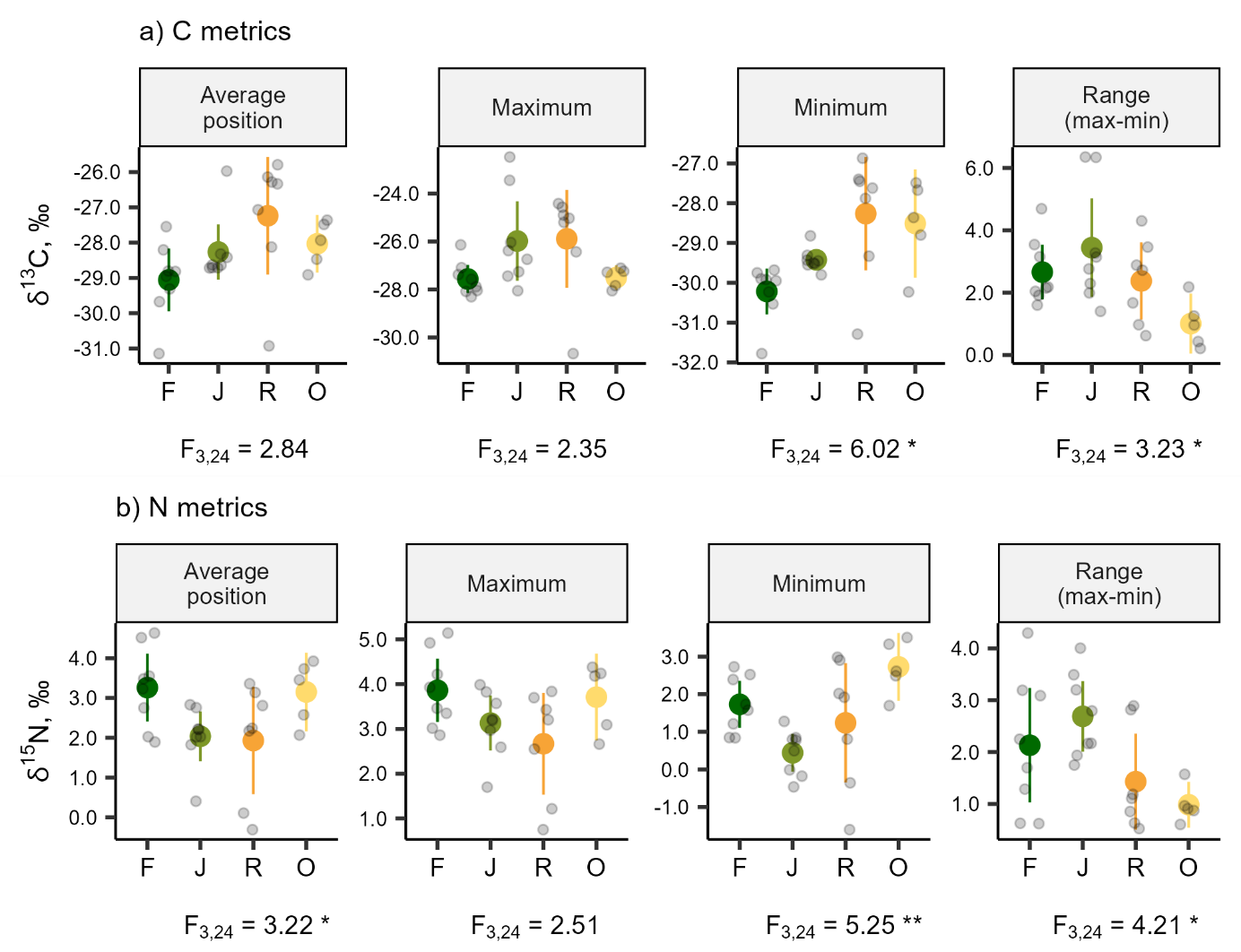


**FIGURE S3 (above).** Abundance-weighted average, maximum, minimum and range of ‘uncalibrated’ ẟ^13^C and ẟ^15^N of 13 species of *Crematogaster* spp. from four land use systems in Jambi, Sumatra, Indonesia (dark green = rainforest, 'F'; green = jungle rubber 'J'; orange = rubber 'R'; yellow = oil palm 'O'; mean ± s.d.). Test statistics are GLM followed by ANOVA. *: p < 0.05; **: p < 0.01; ***: p < 0.001. ‘Uncalibrated’ refers to bulk stable isotope values of animal tissue without subtracting the values of leaves collected from trees and palms in every plot, indicated by ẟ^13^C and ẟ^15^N (‘uncalibrated’) instead of Δ^13^C and Δ^15^N (‘calibrated’). In ^13^C, ‘uncalibrated’ values have somewhat of an inverted pattern compared to ‘calibrated’ ones, with regard to Average position, Maximum and Minimum, but not range (compare Fig. 3a in the main manuscript). In ^15^N, ‘uncalibrated’ and ‘calibrated’ values are very similar for Average position, Maximum and Minimum, and exactly the same for range. This shows the influence of the ‘canopy effect’ for leaf calibration of bulk stable isotopes of ^13^C for systems with a high canopy, such as rainforest and jungle rubber (see also the caption for Fig. S4 and the main text for the explanation of the ‘canopy effect’).


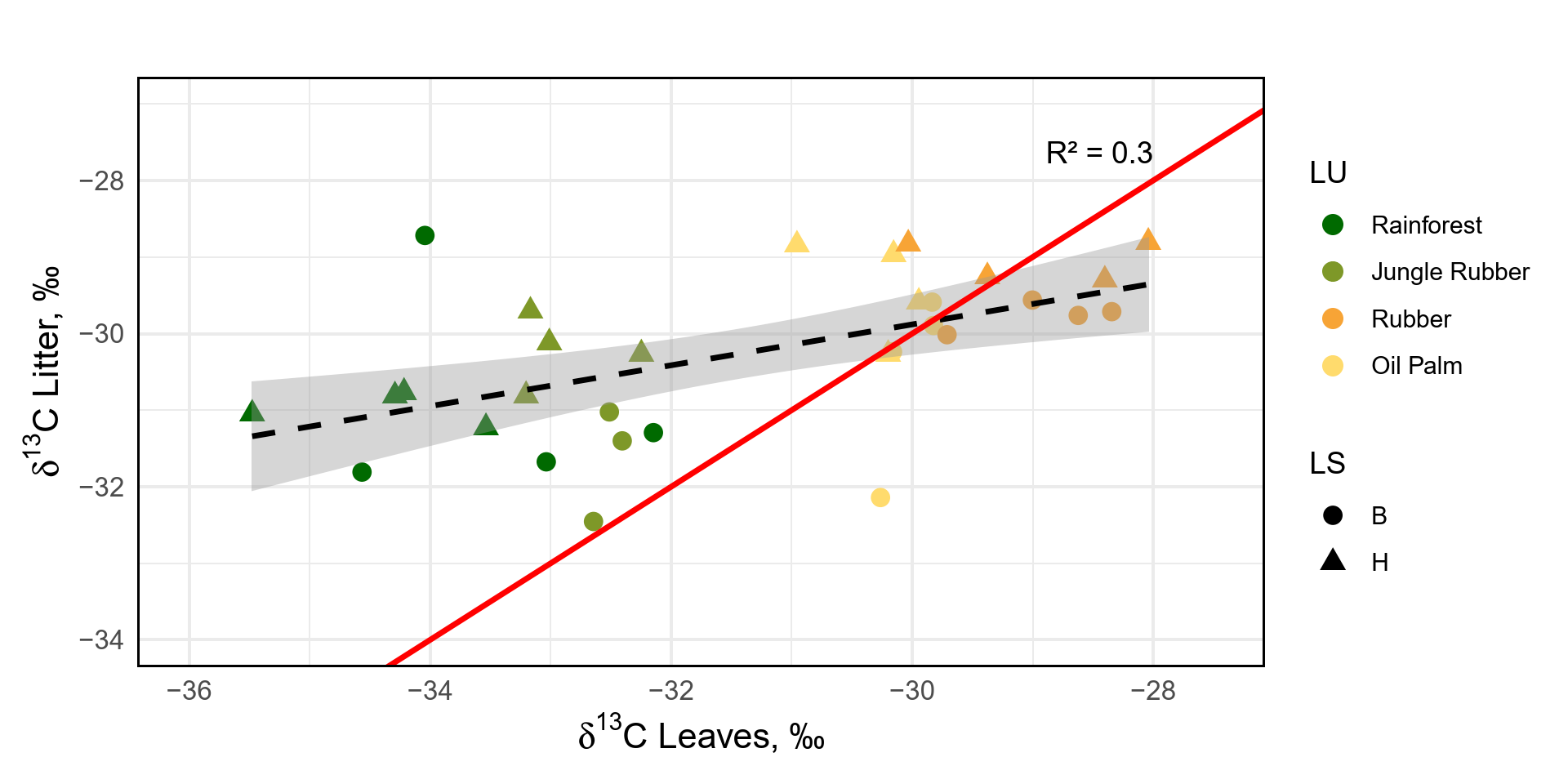


canopy effect

**A**


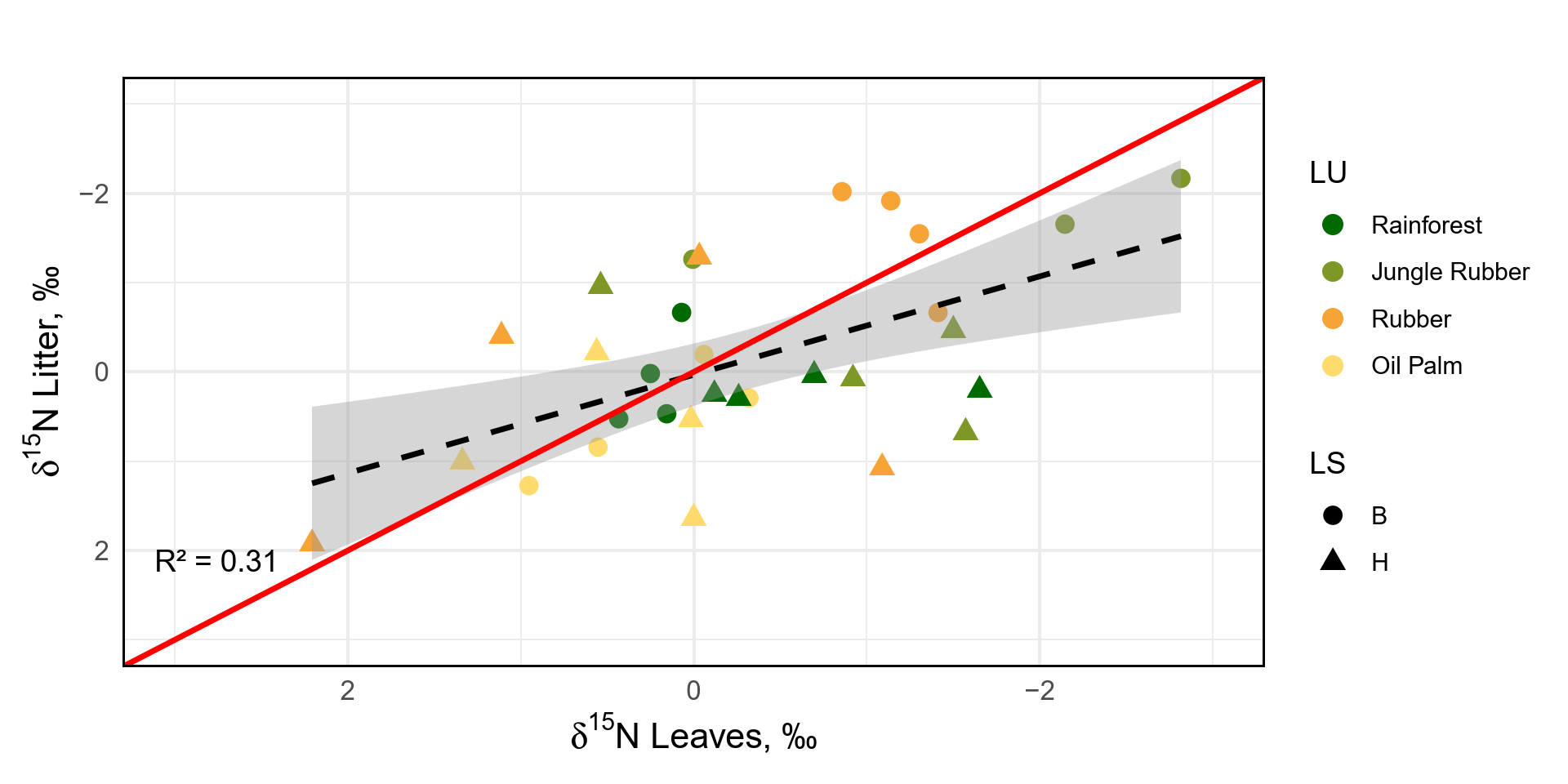


**B**

**FIGURE S4 (above)**

Linear regressions (dotted line) and isocline (red line) between "Leaves" and "Litter" for (A) ẟ^13^C and (B) ẟ^15^N. Five fresh leaves from randomly selected trees near the plot center were collected for "Leaves", while "Litter" data was sourced from Klarner & al. (2017) who measured ẟ^13^C and ẟ^15^N from leaf litter in three soil cores per plot. "Leaves" were from the lower canopy (about 2 m above ground) and more depleted in ẟ^13^C than “Litter” (fallen leaves from lower and higher canopy) in rainforest and jungle rubber due to the “canopy effect”(van der Merwe & Medina 1991). In the “canopy effect”, leaves from lower canopies are more depleted in ^13^C due to photosynthetic recycling of CO_2_ from soil respiration, while leaves from higher canopies acquire more CO_2_ from the surrounding atmosphere, which is less depleted in ^13^C. Accordingly, in the present study, the canopy effect in ^13^C only occurs in rainforest and jungle rubber (dotted ellipse in (A)), partially explaining higher Δ^13^C values of *Crematogaster* acrobat ants in lowland rainforest and jungle rubber and lower values in rubber and oil palm.


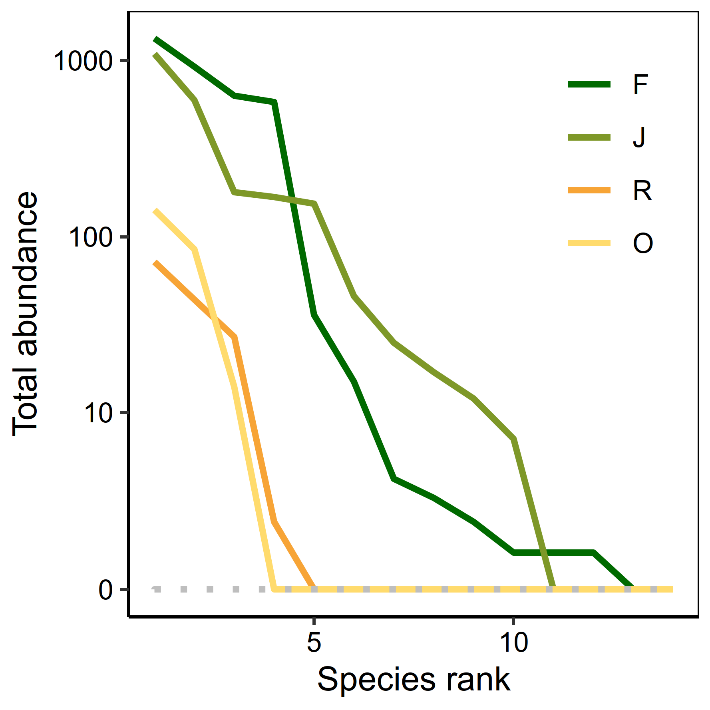


**FIGURE S5 (left).** Ranked abundances of 6211 *Crematogaster* specimens from 14 Linnaean species across four land use systems in Jambi Province, Sumatra, Indonesia (dark green = rainforest, 'F'; green = jungle rubber 'J'; orange = rubber 'R'; yellow = oil palm 'O').


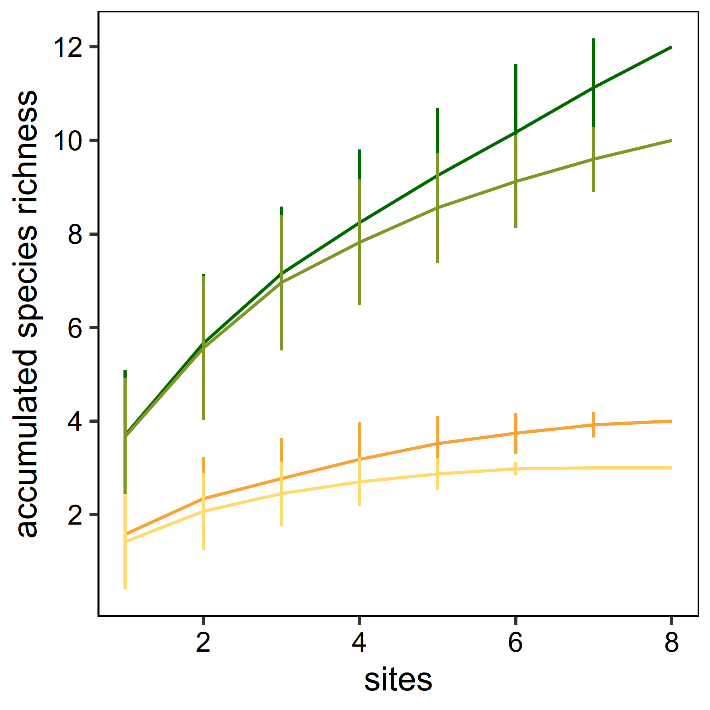


**FIGURE S6 (left).** Species Accumulation curves of 6211 *Crematogaster* specimens from 14 Linnaean species across four land use systems in Jambi Province, Sumatra, Indonesia (dark green = rainforest, 'F'; green = jungle rubber 'J'; orange = rubber 'R'; yellow = oil palm 'O').

**REFERENCES**

Klarner, B., Winkelmann, H., Krashevska, V., Maraun, M., Widyastuti, R. & Scheu, S. 2017: Trophic niches, diversity and community composition of invertebrate top predators (Chilopoda) as affected by conversion of tropical lowland rainforest in Sumatra (Indonesia). – PLoS ONE 12: e0180915.

Merwe, N.J. van der & Medina, E. 1991: The canopy effect, carbon isotope ratios and foodwebs in amazonia. – Journal of Archaeological Science 18: 249-259.

Ramos, D., Hartke, T.R., Buchori, D., Dupérré, N., Hidayat, P., Lia, M., Harms, D., Scheu, S. & Drescher, J. 2022: Rainforest conversion to rubber and oil palm reduces abundance, biomass and diversity of canopy spiders. – PeerJ 10: e13898.


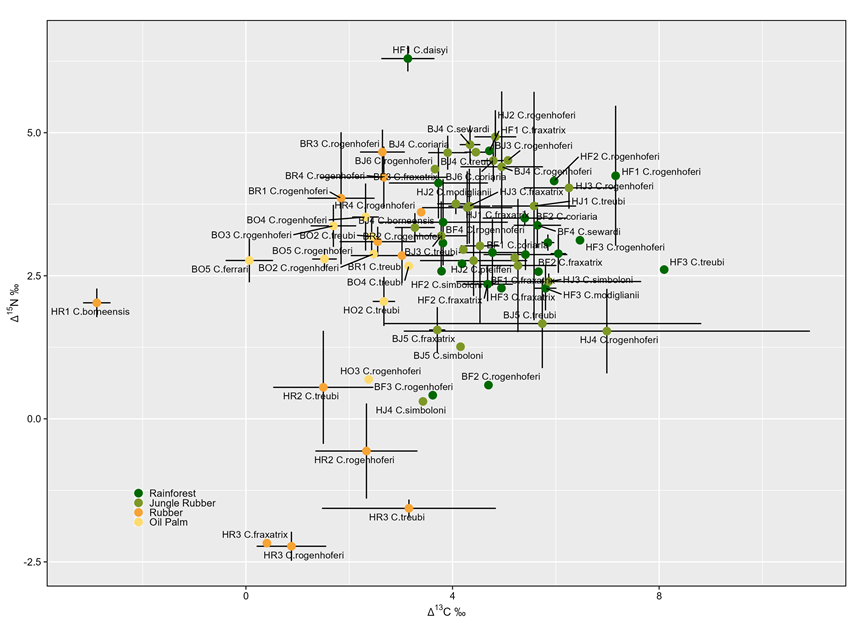


**FIGURE S7.** Biplot of abundance weighted average Δ^13^C and Δ^15^N per plot of 14 species of *Crematogaster* spp. from four land-use systems in Jambi, Sumatra, Indonesia (dark green = rainforest, 'F'; green = jungle rubber 'J'; orange = rubber 'R'; yellow = oil palm 'O'; mean ± s.d.), separated by species and plot. At least four species contribute to the high variation of stable isotope positions of *Crematogaster* communities in rubber and oil palm, i.e. *C. rogenhoferi*, *C. treubi*, *C. fraxatrix* and *C. borneensis*, and this is especially evident in the Harapan landscape (plot names that start with “H”).
